# LiDAR-IMU Time Delay Calibration Based on Iterative Closest Point and Iterated Sigma Point Kalman Filter

**DOI:** 10.3390/s17030539

**Published:** 2017-03-08

**Authors:** Wanli Liu

**Affiliations:** School of Mechanical and Electrical Engineering, China University of Mining and Technology, Xuzhou 221116, China; lwl412101@cumt.edu.cn; Tel.: +86-516-8359-0706

**Keywords:** LiDAR, inertial measurement unit, iterative closest point, iterated sigma point Kalman filter, time delay calibration

## Abstract

The time delay calibration between Light Detection and Ranging (LiDAR) and Inertial Measurement Units (IMUs) is an essential prerequisite for its applications. However, the correspondences between LiDAR and IMU measurements are usually unknown, and thus cannot be computed directly for the time delay calibration. In order to solve the problem of LiDAR-IMU time delay calibration, this paper presents a fusion method based on iterative closest point (ICP) and iterated sigma point Kalman filter (ISPKF), which combines the advantages of ICP and ISPKF. The ICP algorithm can precisely determine the unknown transformation between LiDAR-IMU; and the ISPKF algorithm can optimally estimate the time delay calibration parameters. First of all, the coordinate transformation from the LiDAR frame to the IMU frame is realized. Second, the measurement model and time delay error model of LiDAR and IMU are established. Third, the methodology of the ICP and ISPKF procedure is presented for LiDAR-IMU time delay calibration. Experimental results are presented that validate the proposed method and demonstrate the time delay error can be accurately calibrated.

## 1. Introduction

In today’s world, Light Detection and Ranging (LiDAR) devices and Inertial Measurement Units (IMUs) often found on vehicles, airplanes and robots are increasingly being used to perform localization or for navigation tasks. The LiDAR and IMU sensors, together, can supply accurate attitude estimation and are very suitable in many applications. However, in GPS-denied environments the IMUs are usually unreliable with respect to position for long periods of time due to time drift, which may cause large cumulative errors, the a LiDAR is a device which uses laser beams to determine the distance and azimuth from the sensor to an object, which can provide 3D localization information with high accuracy and efficiency to reduce or bound IMUs drift. Therefore, the integrated LiDAR-IMU system can provide highly accurate position and pose information over long periods of time in GPS-denied environments.

Usually combining information from multiple sensors offers several potential advantages, including enhanced accuracy and improved robustness, but these come at a cost: a fundamental requirement in any multiple sensor system is time delay calibration. To ensure optimal performance, the LiDAR and IMU sensors must be properly time delay calibrated, including estimates of the relative timing of each sensor measurement, coordinate transformation between the different sensors, and time delay calibration parameters estimation are required [1,2,3,4].

Several methods exist for LiDAR, IMU and camera time delay calibration. The work of Kelly et al. [5] presented a time delay iterative closest point method for determining the time delay between inertial and visual sensor measurements. Aghili et al. [6] presented a robust 6-Degree of Freedom (DOF) relative navigation sysetm by combining the iterative closest point (ICP) registration algorithm and a noise Adaptive Kalman Filter (AKF) in a closed loop configuration together with measurements from a LiDAR and an IMU. Pothou et al. [7] investigated the determination of the misalignment between the IMU body frame and the LiDAR frame, using a Quality Assurance/Quality Control (QA/QC) technique to evaluate the LiDAR-IMU bore sight misalignment. Deymier et al. [8] proposed the self-calibration of a vehicle’s acquisition system with cameras, IMU and 3D LiDAR. Jian et al. [9] integrated two complementary technologies—Inertial Navigation System (INS) and LiDAR Simultaneous Localization and Mapping (SLAM)—into one navigation frame with a loosely coupled Extended Kalman Filter (EKF) to use the advantages and overcome the drawbacks of each system to establish a stable long-term navigation process. Mirzaei et al. [10] presented an EKF for precisely determining the unknown transformation between a camera and an IMU. Yun et al. [11] developed the IMU/Vision/LiDAR integrated navigation system which can provide accurate relative navigation information in GNSS-denied environments; and construct an overall integrated navigation filter based on the EKF approach. Li et al. proposed [12] a method of integrating the measurements from a LIDAR and a Micro-Electro-Mechanical System (MEMS) IMU, and using the Kalman Filter (KF) to estimate the error of IMU and LIDAR sensors.

As mentioned above, the AKF, EKF, KF and ICP algorithms are used in LiDAR, IMU and camera sensors to calibrate the time delay. During the course of LiDAR-IMU calibration, neither the EKF algorithm nor AKF algorithm can avoid time delay calibration bias and filtering divergences. Initially, the correspondences between LiDAR and IMU measurements are usually unknown; as a result the relative time delay information between the LiDAR-IMU data flows cannot be computed directly [13,14,15,16]. The question is posed differently as follows: first, the measurement rate information of LiDAR and IMU cannot be directly compared. Second, in general, the rigid body transformation between the LiDAR and the IMU is unknown. Third, both signals are discrete and have different temporal resolutions, the LiDAR typically scanning information is available at 5–20 Hz or less, however the IMU produces data is normally available at 100 Hz or more [17,18,19,20].

In order to solve the problem of LiDAR-IMU time delay calibration, we present a fusion method based on ICP and an iterated sigma point Kalman filter (ISPKF), which combines the advantages of ICP and ISPKF [21,22,23]. The total least squares cost function is used by the ICP algorithm for registration, which allows us to merge the IMU orientation measurement uncertainty in a principled way, and to reduce the longer time intervals due to the accumulated noise effects by integrated LiDAR orientation measurement. The ISPKF algorithm measurement update step is iterated until the change in the posterior state estimate drops below some small threshold, and can optimally estimate the time delay calibration parameters. First of all, the coordinate transformation from the LiDAR frame to the IMU frame is realized. Second, the measurement model and time delay error model of LiDAR and IMU sensors are established. Third, the methodology of the ICP and ISPKF procedure is presented for LiDAR-IMU time delay calibration. Experimental results are presented that validate the proposed method and demonstrate the time delay can be accurately calibrated.

This paper is organized as follows: the coordinate transformation between LiDAR and IMU are described in Section 2. In Section 3, we establish the LiDAR and IMU measurement models and the time delay error model in LiDAR-IMU. Section 4 given the implementation procedure of ICP-ISPKF for LiDAR-IMU time delay calibration. Section 5 experimental studies and analysis the results using LiDAR, IMU and optical measuring system based our proposed method are described, followed by the conclusions in Section 6.

## 2. Coordinate Transformation between LiDAR and IMU

### 2.1. Coordinate Frame

**Figure 1:**
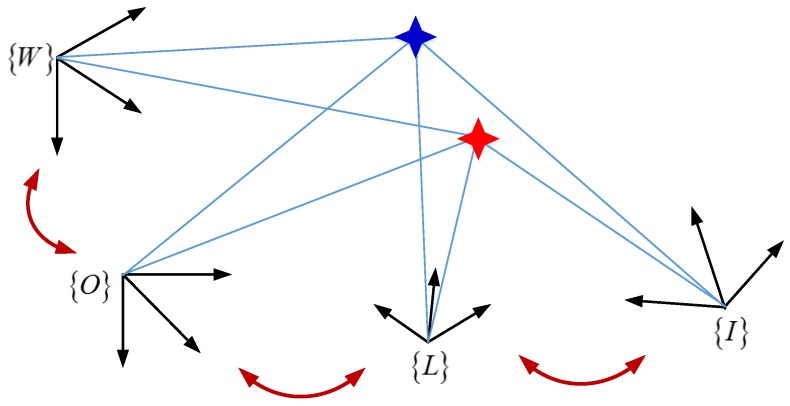
Relationship between the {*L*}, {*I*}, {*O*} and {*W*}.

In general, there are basically four coordinate frames in the LiDAR and IMU, The relationship between these frames is shown in Figure 1:

(1) LiDAR frame, {*L*}, is represented in this frame of reference, in which the axes are defined as right, forward and up.
(2) IMU frame, {*I*}, is defined by the IMU, in which angular rotation rates and linear accelerations are measured, with its origin at a point on the IMU body.
(3) Object frame, {*O*}, is the coordinate of moving object, the axes in the object frame are forward, right and down.
(4) World frame, {*W*}, is considered to be the fundamental coordinate frame and serves as an absolute reference for both the {*I*} and the {*L*}.

### 2.2. Transformation from LiDAR Frame to IMU Frame

#### 2.2.1. Transformation from LiDAR Frame to the World Frame

Suppose a point *^W^P* in the {*W*} frame is located at *^L^P* in the {*L*}, the transformation from the {*W*} coordinate to the {*L*} coordinate system can be expressed as [24]:

(1) $${}^{L}P=R_{W}^{L}{}^{W}P+{}^{L}T_{LW}$$

where $R_{W}^{L}$ is a 3 × 3 orthonormal matrix representing the rotation from the {*W*} frame to the {*L*} frame, and ${}^{L}T_{LW}$ is the translation vector. The subscript is the origin of the {*L*} coordinate, and is the origin of the tangent frame. Our goal is to calculate $R_{W}^{L}$ and ${}^{L}T_{LW}$.

The transformation is implemented through the observation of LiDAR scanning to a calibration plane. A geometric constraint can be obtained between LiDAR scanning points and the calibration plane. Since LiDAR scanning points lie on the calibration plane, and *^W^r* is the normal vector to the plane, we have:

(2) $${}^{W}r\cdot {}^{W}P=d$$

where *^W^P* is the coordinate of LiDAR scanning point in {*W*} frame; *d* is a scalar representing the distance from the origin of the {*W*} frame to the calibration plane, which is calculated from the position and orientation of the calibration plane. From Equation (1) we know that:

(3) $${}^{W}P={\left(R_{W}^{L}\right)}^{-1}\left({}^{L}P-{}^{L}T_{LW}\right)$$

By substituting Equation (3) into Equation (2), we have:

(4) $${}^{W}r\cdot {\left(R_{W}^{L}\right)}^{-1}\left({}^{L}P-{}^{L}T_{LW}\right)=d$$

For any given LiDAR scanning point and calibration plane position, Equation (4) gives a constraint on $R_{W}^{L}$ and ${}^{L}T_{LW}$. It will be solved in two consecutive steps: a linear solution, followed by a non-linear optimization [25,26]:

(1) Linear solution. The LiDAR scanning plane in the {*L*} is ${}^{L}Z=0$. Each LiDAR scanning point can be represented as ${}^{L}P={\left[{}^{L}P_{x}\;{}^{L}P_{y}\;{}^{L}P_{z}\right]}^{T}$. Then Equation (4) is rewritten as:

(5) $${}^{W}r\cdot {\left(R_{W}^{L}\right)}^{-1}\left(\left[\begin{array}{c} {}^{L}P_{x}\\ {}^{L}P_{y}\\ {}^{L}P_{z} \end{array}\right]-{}^{L}T_{LW}\right)={}^{W}r\cdot {\left(R_{W}^{L}\right)}^{-1}\left(\left[\begin{array}{ccc} 1 & 0 & 0\\ 0 & 1 & 0\\ 0 & 0 & 1 \end{array}\right]-{}^{L}T_{LW}\right)\left[\begin{array}{c} {}^{L}P_{x}\\ {}^{L}P_{y}\\ {}^{L}P_{z} \end{array}\right]=d$$

Let’s define $Z={\left(R_{W}^{L}\right)}^{-1}\left(\left[\begin{array}{ccc} 1 & 0 & 0\\ 0 & 1 & 0\\ 0 & 0 & 1 \end{array}\right]-{}^{L}T_{LW}\right)$, Equation (5) is rewritten as:

(6) $${}^{W}r\cdot Z\cdot {}^{L}P=d$$

where *Z* is the parameter to be solved, which is the integration of two unknown parameters $R_{W}^{L}$ and ${}^{L}T_{LW}$.

Equation (6) can be solved using the standard linear least squares algorithm. In order to obtain a better result, multiple calibration planes should be used in the transformation. Suppose in the transformation we use a total of *N* calibration planes, with *M_i_* (*i* = 1,…,*N*) LiDAR scanning points on the *i*-th plane. Let $Z=\left[\begin{array}{ccc} z_{11} & z_{12} & z_{13}\\ z_{21} & z_{22} & z_{23}\\ z_{31} & z_{32} & z_{33} \end{array}\right]$, the normal vector for the *i*-th plane ${}^{W}r_{i}=\left[r_{i,1}\;r_{i,2}\;r_{i,3}\right]$, the distance from origin of the {*W*} frame and the *i*-th calibration plane is *d_i_* and the *j*-th LiDAR scanning point on the *i*-th calibration plane is ${}^{L}P_{ij}={\left[{}^{L}P_{ij,x}\;{}^{L}P_{ij,y}\;{}^{L}P_{ij,z}\right]}^{T}$, Equation (6) is rewritten as:

(7) $$(r_{i,1}z_{11}+r_{i,2}z_{21}+r_{i,3}z_{31})\cdot {}^{L}P_{ij,x}+(r_{i,1}z_{12}+r_{i,2}z_{22}+r_{i,3}z_{32})\cdot {}^{L}P_{ij,y}+(r_{i,1}z_{13}+r_{i,2}z_{23}+r_{i,3}z_{33})\cdot {}^{L}P_{ij,z}=d_{i}$$

where *i* = 1,2,…,*N*, *j* = 1,2,…,*M*.

Therefore for each LiDAR scanning point we have a linear equation which is a row in Equation (7) can be calculated using the standard linear least square algorithm. Then $R_{W}^{L}$ and ${}^{L}T_{LW}$ will be obtained from *Z*.

(2) Nonlinear solution. The linear solution is obtained by the least squares method, which aims to minimize an algebraic distance. A nonlinear minimization method is used to minimize the differences between the measured Euclidean distances as well as the calculated distance from the LiDAR scanning points to the calibration plane, which is physically meaningful.

Equation (4) gives two types of distances: *d* is the distance from the calibration plane to the origin of the {*W*} frame obtained by a field survey, and ${}^{W}r\cdot {\left(R_{W}^{L}\right)}^{-1}({}^{L}P-{}^{L}T_{LW})$ is the calculated distance. The difference between these two distances is defined as the distance error for one calibration plane pose. The nonlinear solution aims to minimize the sum function of the distance errors for all the plane positions. The sum function is defined as:

(8) $$sum=\sum_{i=1}^{N}{\sum_{j=1}^{M_{i}}\left({}^{W}r_{i}\cdot {\left(R_{W}^{L}\right)}^{-1}\left({}^{L}P_{ij}-{}^{L}T_{LW}\right)-d_{i}\right)}^{2}$$

where *ri* defines the *i*-th calibration plane, *d_i_* is the distance from the *i*-th plane to the center of the {*W*} frame, and *P_ij_* is the *j*-th LiDAR scanning point with the *i*-th calibration plane; the pair of $R_{W}^{L}$ and ${}^{L}T_{LW}$ that minimize Equation (8) are considered to be the rotation and translation matrix to be calculated. Equation (8) can be minimized as a nonlinear optimization problem by getting the translation and rotation matrix between the {*W*} frame and LiDAR scanning points in the {*W*} frame can be converted to a point in the {*L*} frame.

#### 2.2.2. Transformation from LiDAR Frame to IMU Frame

The transformation of the LiDAR frame to the IMU one is to obtain the geometric relationship between the {*L*} frame and {*O*} frame. The IMU consists of three gyros and three accelerometers. The gyros provide change of Euler angles, while the accelerometers give the specific force. By integrating the output of the gyros and the accelerometers, we can obtain the translation and rotation matrix between the {*W*} frame and the {*O*} frame. Let the rotation matrix be $R_{O}^{W}$, and translation vector be ${}^{O}T_{WO}$, then a point in the {*O*} frame can be converted to a point in the {*W*} frame by:

(9) $${}^{W}P=R_{O}^{W}{}^{O}P+{}^{O}T_{WO}$$

where ${}^{W}P$ is the point in the {*W*} frame, and ${}^{O}P$ is the point in the {*O*} frame.

Finally, by substituting Equation (1) into Equation (9) we have:

(10) $${}^{L}P=R_{W}^{L}\left(R_{O}^{W}{}^{O}P+{}^{O}T_{WO}\right)+{}^{L}T_{LW}$$

Equation (10) is the transformation from the {*O*} frame to the {*L*} frame.

## 3. LiDAR and IMU Measurement Model

### 3.1. LiDAR Measurement Model

**Figure 2:**
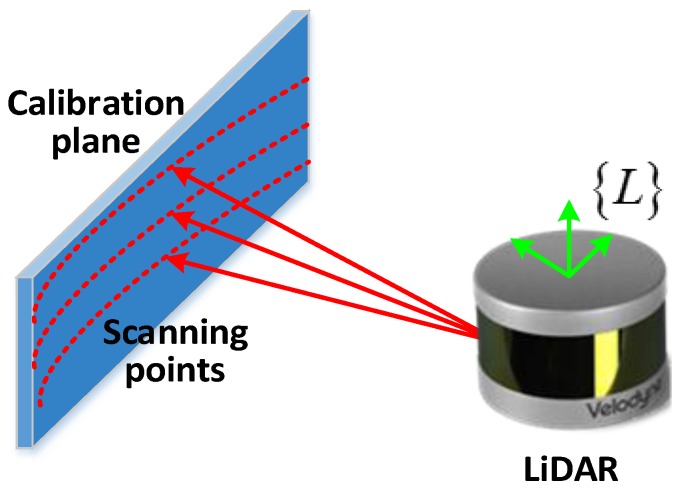
The LIDAR position and point feature detection.

The LiDAR scans the laser beam through 360°. Therefore, a post which will be modeled as a vertical line in the tangent frame will appear as a point in the LiDAR frame. Similarly, a wall which will be modeled as a vertical plane in the tangent frame will appear as a line in the LiDAR frame of references. The LiDAR position and point feature detection are shown in Figure 2.

Consider a mapped vertical post at *P*_0_. The post will appear as a point in the LiDAR return. The vector from *T* to *P*_0_ in world frame is ${}^{W}T_{LP_{O}}={\left[N_{0}\;E_{0}\;0\right]}^{T}$, which is known from the survey. The line is denoted as:

(11) $${}^{W}T_{WP}(m)={}^{W}T_{WP_{O}}+me_{3}$$

where ${}^{W}T_{WP}(m)$ is the vector in world frame from *T* to a point *P* at height *m* on the post, *m* is a scalar with m∈(0,+∞). The vector *e*_3_ is parallel to the post. In our application, all the posts and planes are modeled as vertical. For a vertical post $e_{3}={\left[0\;0\;1\right]}^{T}$ in the world frame.

For any feature point *P*, the vector from the LiDAR to the point is:

(12) $$T_{LP}=T_{WP}-(T_{WO}+T_{OL})$$

**Figure 3:**
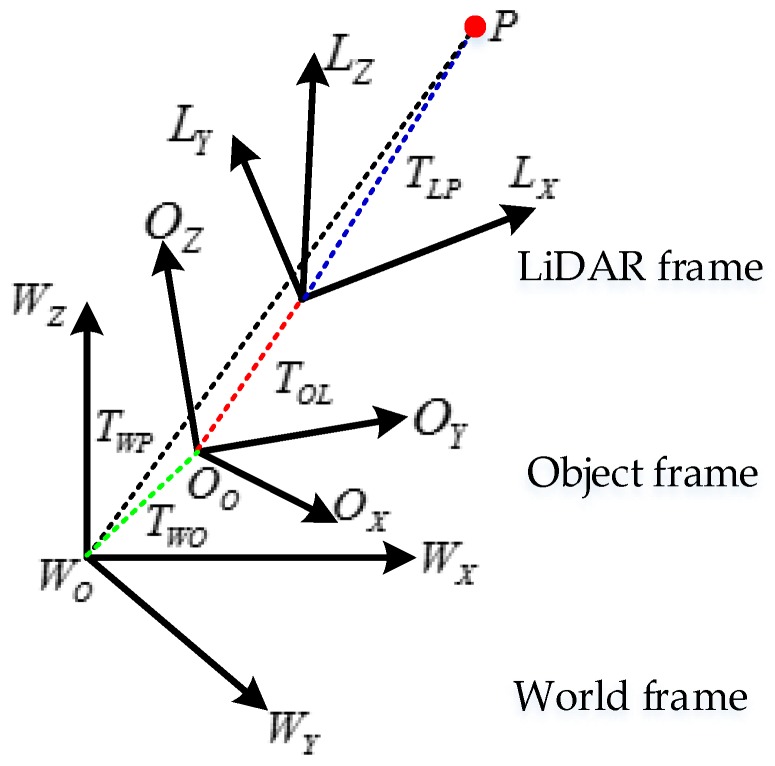
The LiDAR, Object and World frames as well as the feature point *P*.

As is shown in Figure 3. Equation (12) is valid for all the reference frames.

In Equation (12) and Figure 3, *T_WP_* is a constant in the world frame known from the feature survey. *T_WO_* will be calculated in the world frame. *T_OL_* in the object frame is known, and can be determined by the pre-calibration.

$R_{O}^{L}$ is the rotation matrix from the object frame to the LiDAR frame, so the vector from the LiDAR to a feature point in LiDAR coordinates is denoted as:

(13) $${}^{L}T_{LP}=R_{O}^{L}\left(R_{W}^{O}({}^{W}T_{WP}-{}^{W}T_{WO})-{}^{O}T_{OL}\right)$$

By substituting Equation (11) into Equation (13) we have:

(14) $${}^{L}T_{LP}(m)=R_{O}^{L}\left(R_{W}^{O}({}^{W}T_{WP}+me_{3}-{}^{W}T_{WO})-{}^{O}T_{OL}\right)$$

which is the vector in the LiDAR coordinates from the origin of the LiDAR sensor to a point with height *m* on the post. By expanding Equation (14), the coordinate of ${}^{L}T_{LP}\left(m\right)$ in the $-{}^{L}Z$ direction is:

(15) $$Z(m)=e_{3}^{W}\cdot {}^{L}T_{LP}(m)=m\cdot e_{3}^{W}\cdot R_{O}^{L}\cdot R_{W}^{O}\cdot e_{3}+e_{3}^{W}\cdot R_{O}^{L}\left(R_{W}^{O}({}^{W}T_{WP}-{}^{W}T_{WO})-{}^{O}T_{OL}\right)$$

Equation (15) is the theoretical equation. Note that for a single-planar LiDAR, the scan plane in LiDAR coordinate is ${}^{L}Z=0$. Therefore, for all the LiDAR points we have ${}^{L}Z=0$, and we can use this fact to calculate *m* as:

(16) $$m=-\frac{e_{3}^{W}\cdot R_{O}^{L}\left(R_{W}^{O}({}^{W}T_{WP}-{}^{W}T_{WO})-{}^{O}T_{OL}\right)}{e_{3}^{W}\cdot R_{O}^{L}\cdot R_{W}^{O}\cdot e_{3}}$$

By substituting Equation (16) into Equation (14), the detected point is:

(17) $${}^{L}T_{LP}(m)=R_{O}^{L}\left(R_{W}^{O}({}^{W}T_{WP}-\frac{e_{3}^{W}\cdot R_{O}^{L}\left(R_{W}^{O}({}^{W}T_{WP}-{}^{W}T_{WO})-{}^{O}T_{OL}\right)}{e_{3}^{W}\cdot R_{O}^{L}\cdot R_{W}^{O}\cdot e_{3}}e_{3}-{}^{W}T_{WO})-{}^{O}T_{OL}\right)$$

Equation (17) is the LiDAR measurement model.

### 3.2. IMU Measurement Model

In general, the IMU measures the moving object’s rotational motion in three orthogonal axes. Usually, the output rates of IMU measuring angles are not zero, even when the IMU is stationary, and during the IMU measurements the angular products are easily be corrupted by noise. At time *t*, the IMU measuring angular velocity can be written as [5,27]:

(18) $$\omega_{m}(t)=\omega^{I}(t)+b_{g}+n_{g}(t)$$

where $n_{g}\left(t\right)$ is the noise vector, *b_g_* and $\omega^{I}\left(t\right)$ are the bias vector and the true angular velocity of the IMU, respectively.

In fact, all IMUs are subject to time drift or their measurement bias values slow change with time. Usually, in the very short period of time of the calibration interval for the IMU, the measurement bias and drift may be negligible, however, in order to obtain an accurate estimate of the gyroscope biases, at the start of calibration, we average several seconds of IMU data.

At a time *t* > *t*_0_, in order to obtain the IMU orientation estimate, according to the modified Rodrigues parameters kinematic differential equation, we integrate each IMU measurement forward as:

(19) $$\dot{\rho }(t)=\frac{1}{4}\left(\left(1-{\left\|\rho (t)\right\|}^{2})I_{3}+2{\left[\rho (t)\right]}_{\times }+2\rho (t\right)\right)\omega^{I}(t)$$

where $\omega^{I}\left(t\right)=\omega_{m}\left(t\right)-b_{g}-n_{g}\left(t\right)$.

As integration continues, the noise and drift will cause inaccuracies, therefore, relative to its initial orientation, the IMU true orientation will become less certain. By estimating the IMU propagation orientation covariance, the matrices *F* and *G* can be defined as:

(20) $$\left\{ \begin{array}{l} F=\frac{\partial \dot{\rho }}{\partial \upsilon }=\frac{1}{2}\left((\rho^{T}\cdot \omega )I_{3}-{\left[\omega \right]}_{\times }+\rho \omega^{T}-\omega \cdot \rho^{T}\right)\\ G=\frac{\partial \dot{\rho }}{\partial n_{g}}=-\frac{1}{4}\left((1-{\left\|\rho \right\|}^{2})I_{3}+2{\left[\rho \right]}_{\times }+2\rho \rho^{T}\right) \end{array}\right.$$

Using the continuous time equation the propagation orientation covariance can be written as:

(21) $${\dot{P}}_{I}=FP_{I}+P_{I}F^{T}+GQ_{g}G^{T}$$

According to Equation (21), any feature point in the IMU measurement curve can be selected as a zero uncertainty reference. We choose one of the earliest IMU measurements as the uncertainty computation reference, mainly because our computed deviation estimate is relative to the earliest measurement.

### 3.3. Time Delay Error Model

We will identify a specific instantaneous local LiDAR frame as {*L_k_*} with timestamp $t_{L_{k}}$ according the receiver clock, for *k* = 1,…,*n* LiDAR poses. Similarly, for *j* = 1,…,*m* IMU poses, the {*I_j_*} will be identified as a specific instantaneous local IMU frame with timestamp $t_{I_{j}}$. In general, the IMU data are available at a substantially higher rate than the LiDAR data, and *m* > *n*.

To compare LiDAR and IMU measurement data, a common representation is required; we use the orientation measurements to match IMU and LiDAR. The IMU orientation is measured with respect to the initial sensor pose. The LiDAR orientation is measured relative to a series of calibration planes. Our algorithm employs the modified Rodrigues parameters as a minimal and convenient orientation representation. The use of the modified Rodrigues parameters vector representation allows us to propagate the IMU orientation uncertainty forward continuously in time [28].

We have the following relationship at any time $t_{I_{j}}$:

(22) $${}_{I}^{W}\rho (t)={}_{L}^{W}\rho (t+\tau )\cdot {}_{I}^{L}\rho$$

where ${}_{I}^{L}\rho$ is the modified Rodrigues parameter vector with the orientation from the {*I*} frame to the {*L*} frame. ${}_{L}^{W}\rho \left(t+\tau \right)$ is the modified Rodrigues parameter vector with the orientation from the {*L*} frame to the {*W*}, τ is the time delay. ${}_{L}^{W}\rho \left(t\right)$ is the modified Rodrigues parameter vector with the orientation from the {*I*} frame to the {*W*} frame.

Although the orientation of the rate measurement information supplied by the IMU cannot be measured directly, by integrating the IMU gyroscope data, we can obtain the IMU measurement orientation change over a period of time. Similarly, we can conveniently compute the LiDAR measured orientation relative to the {*W*} frame. The measurement relationship between the {*I*}, {*L*} and {*W*} frame can be computed as:

(23) $${}_{I}^{I_{0}}\rho (t)={}_{W}^{I_{0}}\rho \cdot {}_{L}^{W}\rho (t+\tau )\cdot {}_{I}^{L}\rho$$

where ${}_{W}^{I_{0}}\rho$ is the modified Rodrigues parameter vector with the orientation from the {*I*_0_} frame to the {*W*} frame.

(1) Position error analysis: the error in position can be calculated as follows:

(24) $$\delta \left({}^{W}\dot{T}{}_{WO}\right)=R_{O}^{W}\cdot {}^{O}V-{\hat{R}}_{O}^{W}\cdot {}^{O}\hat{V}={\hat{R}}_{O}^{W}\cdot \delta \left({}^{O}V\right)-{}^{W}\hat{V}\cdot \rho$$

where $R_{O}^{W}={\hat{R}}_{O}^{W}\left(I+\rho \right),\;I={\left(R_{W}^{L}\right)}^{T}\cdot R_{W}^{L},\;\rho$ is the calibration plane tilt error, and ${}^{O}V={}^{O}\hat{V}+\delta \left({}^{O}V\right)$.

(2) Velocity error analysis: the error in velocity is modeled as follows:

(25) $$\begin{array}{ll} \delta \left({}^{O}\dot{V}\right) & ={}^{O}\dot{V}-{}^{O}\dot{\hat{V}}={}^{O}a_{O}-\left(\Omega_{IL}^{O}+\Omega_{IO}^{O}\right)\cdot {}^{O}V-{}^{O}{\hat{a}}_{O}+\left({\hat{\Omega }}_{IL}^{O}+{\hat{\Omega }}_{IO}^{O}\right)\cdot {}^{O}\hat{V}\\ & ={\hat{R}}_{O}^{W}\left(\left(\frac{\partial \left(g^{W}\right)}{\partial \left({}^{W}T_{WO}\right)}+{}^{W}\hat{V}\cdot \frac{\partial \left({}^{W}\omega_{IL}\right)}{\partial \left({}^{W}T_{WO}\right)}\right)\delta \left({}^{W}T_{WO}\right)+\left(g^{W}+{}^{W}\omega_{IL}{\left({}^{W}\hat{V}\right)}^{T}-{\left({}^{W}\omega_{IL}\right)}^{T}\cdot {}^{W}\hat{V}\cdot I\right)\rho \right)\\ & -\left({\hat{\Omega }}_{IL}^{O}+{\hat{\Omega }}_{IO}^{O}\right)\delta \left({}^{O}V\right)-\delta b_{a}-{}^{O}\hat{V}\cdot \delta b_{g}-\gamma_{a}-{}^{O}\hat{V}\cdot \gamma_{g} \end{array}$$

where *g^W^* is the local gravity vector at the moving object location represented in the {*W*} frame, ${}^{W}\omega_{IL}$ is the angular rate of the {*W*} frame origin to the {*I*} frame represented in the {*L*} frame. $\gamma_{g}$ is the white Gaussian measurement noise, *b_g_* is the gyro bias, which is modeled as a random constant plus random noise.

(3) Attitude error analysis: the attitude error is given by:

(26) $$\delta \left(\dot{\Theta }\right)=\dot{\Theta }-\dot{\hat{\Theta }}=\Omega_{E}^{-1}\cdot {}^{W}{\hat{\omega }}_{IW}-\delta \left({}^{W}\omega_{IW}\right)-{\hat{R}}_{I}^{W}\left(\delta b_{g}+\gamma_{g}\right)$$

where $\delta ({}^{W}\omega_{IW})$ can be calculated as:

(27) $$\delta \left({}^{W}\omega_{IW}\right)=-\omega_{IL}\cdot \left[\begin{array}{c} \sin\alpha \\ 0\\ \cos\alpha \end{array}\right]\cdot \frac{\partial \Theta }{\partial \left({}^{W}R_{IW}\right)}\cdot \delta \left({}^{W}R_{IW}\right)$$

(4) Time delay calibration parameters error model: the measurement noise vector, the time delay parameter vector and process noise vector of LiDAR-IMU can be expressed as:

(28) $$\left\{ \begin{array}{l} x={\left[\begin{array}{ccccc} \delta \left({}^{W}T_{WO}\right) & \delta \left({}^{O}V\right) & \delta \Theta & \delta \omega_{ba} & \delta \omega_{bg} \end{array}\right]}^{T}\\ b={\left[\begin{array}{cc} b_{a} & b_{g} \end{array}\right]}^{T}\\ \gamma ={\left[\begin{array}{cc} \gamma_{a} & \gamma_{g} \end{array}\right]}^{T} \end{array}\right.$$

where the *x*, *b* and *γ* are the time delay of the error state vector, process noise vector and measurement noise vector, respectively.

According to the Equations (25)–(28), the time delay calibration parameters error model of LiDAR-IMU can be computed as:

(29) $$\left[\begin{array}{c} \delta \left({}^{W}{\dot{T}}_{WO}\right)\\ \delta \left({}^{O}\dot{V}\right)\\ \delta \dot{\Theta }\\ \delta {\dot{\omega }}_{ba}\\ \delta \omega_{bg} \end{array}\right]=\left[\begin{array}{ccccc} {\hat{R}}_{O}^{W}\frac{\partial \left(g^{W}\right)}{\partial \left({}^{W}T_{WO}\right)} & {\hat{R}}_{O}^{W} & -{}^{W}\hat{V} & 0 & 0\\ 0 & -\left({\hat{\Omega }}_{IL}^{O}+{\hat{\Omega }}_{IO}^{O}\right) & -{\hat{R}}_{O}^{W}g^{W} & -I & -{}^{O}\hat{V}\\ 0 & 0 & {\hat{\Omega }}_{E}^{-1} & 0 & -{\hat{R}}_{O}^{W}\\ 0 & 0 & 0 & 0 & 0\\ 0 & 0 & 0 & 0 & 0 \end{array}\right]\cdot \left[\begin{array}{c} \delta \left({}^{W}T_{WO}\right)\\ \delta \left({}^{O}V\right)\\ \delta \Theta \\ \delta \omega_{ba}\\ \delta \omega_{bg} \end{array}\right]+\left[\begin{array}{cccc} -I & 0 & 0 & 0\\ 0 & -{}^{O}\hat{V} & 0 & 0\\ 0 & -{\hat{R}}_{O}^{W} & 0 & 0\\ 0 & 0 & I & 0\\ 0 & 0 & 0 & I \end{array}\right]\cdot \left[\begin{array}{c} \gamma_{a}\\ \gamma_{g}\\ b_{a}\\ b_{g} \end{array}\right]$$

## 4. Time Delay Calibration Using the ICP-ISPKF Integration Method

The ICP algorithm can be used to register the spatial data from IMU and LiDAR measurements, which operates by aligning two curves in a 3-D orientation space generated from integrated IMU gyroscope data and from LiDAR scanner data. Each point on the respective curve has a corresponding timestamp, identifying the time at which the measurement arrived at the receiver. By registering the orientation curves, we are able to use the timestamp values to estimate the relative delay between IMU and LiDAR data streams. ICP incorporates in a principled way the uncertainty in the LiDAR orientation measurements and accounts for the fact that the integrated IMU orientation becomes less accurate over longer intervals due to the incorporation of noise. We specifically avoid this using the ISPKF because of The ISPKF algorithm measurement update step is iterated until the change in the posterior state estimate drops below some small threshold, and can optimally estimate the time delay calibration parameters. The ISPKF is shown to achieve superior performance in terms of efficiency and accuracy compared with the KF, EKF and UKF, also the Gabe Sibley et al. [29] have compared the ISPKF with KF, EKF, UKF and Gauss-Newton filter, and demonstrate the ISPKF’s capabilities in avoiding IMU measuring noise. Therefore, in order to solve the problem of LiDAR-IMU time delay calibration, we use the fusion method based on ICP and ISPKF algorithm.

**Figure 4:**
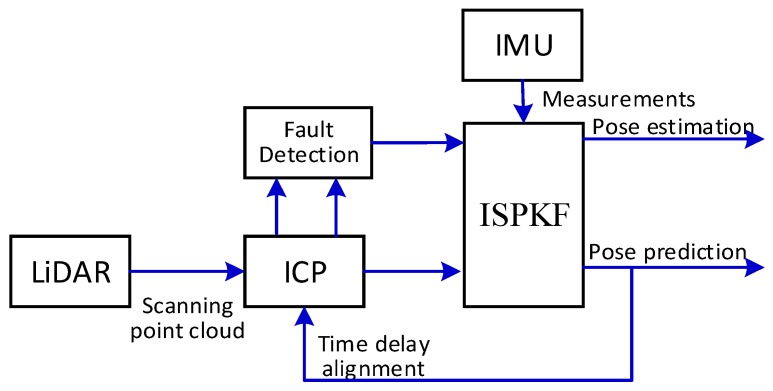
The Architecture of ICP and ISPKF Integration method.

As shown in Figure 4, the LiDAR-IMU time delay calibration is integrated with ICP and ISPKF method. Firstly, once the ICP succeeds matching the LiDAR measurement points, the LiDAR-IMU state estimations are updated with the precise registration value. Secondly, at the time delay filter estimate step, the obtained predicted pose is required to find the corresponding points between LiDAR and IMU measurements. Thirdly, at each time step, the recursive algorithm is used to update the related covariance estimates and precise registration value for LiDAR-IMU. The ICP convergence information and the fault-detection logic are used to adaptively adjust ISPKF reliable estimate of motion state and a set of related parameters. In closed-loop ICP-ISPKF architecture, through ICP initial guess and fault detection, the LiDAR-IMU robust pose tracking and automatic fault recovery are established. Finally, the pose prediction information used to align the time delay error for ICP [6,30].

### 4.1. The ICP Algorithm for Estimation the Time Delay and Relative Orientation of LiDAR-IMU

The ICP algorithm is utilized to estimation the LiDAR-IMU time delay and relative orientation. At the beginning, the transforms between the LiDAR-IMU orientation curves are computed through iteratively selecting n correspondences point using ICP algorithm. We will adjust the search the corresponding time scale with the orientation curves converge. The ICP algorithm can be describe in two steps as follows [5,31]:

Step I: Registration Rules.

The ICP algorithm operates by iteratively selecting the closest point between the IMU orientation curve and the LiDAR measurement point, and the concept of ICP proximity requires a suitable distance measurement.

For the LiDAR measurement point $P_{L_{i}}^{W}$, the distance to the nearby IMU point $P_{I_{j}}^{W}$ is determined by applying the current spatial transformation model and calculating the incremental modified Rodrigues parameter vector. Taking from $P_{L_{i}}^{W}$ to the orientation $P_{I_{j}}^{W}$, we can get the following equation:

(30) $$\sigma_{ij}=-({\hat{\rho }}_{W}^{I_{0}}\cdot \rho_{L_{i}}^{W}\cdot {\hat{\rho }}_{I}^{L})\cdot \rho_{I_{j}}^{I_{0}}$$

According to the Equation (30), it can be known that the closest point is the IMU measurement point, and it produces the minimum value of distance function that can be computed as:

(31) $$d_{ij}=4\arctan\sqrt{\sigma_{ij}^{T}\sigma_{ij}}$$

where *d_ij_* is the incremental rotation arc length taking from $\rho_{L_{i}}^{W}$ to $\rho_{I_{j}}^{I_{0}}$ in radians orientation.

The search ranges of each LiDAR scanning point in the IMU measurement curve are constrained by the maximum expected time delay, and can be definitely expressed as $\left[t_{L_{i}}-\hat{\tau }-\delta t,t_{L_{i}}-\hat{\tau }+\delta t\right]$, where ±δt is the maximum value. When the algorithm converges, the δt can be reduced on each iteration.

Step II: Nonlinear Iterative Registration.

We can get *n* correspondence relationships between the IMU and LiDAR orientation measurement curves by selecting the closest IMU point for each LiDAR point. The generalized nonlinear total least squares method is used to align the IMU and LiDAR orientation curves, where we minimize the cost function as follows:

(32) $$U\left({}_{W}^{I_{0}}\rho ,{}_{I}^{L}\rho \right)=\sum_{k=1}^{n}s_{k}P_{L_{k}}^{-1}s_{k}^{T}+\sum_{k=1}^{n}t_{k}P_{I_{f(k)}}^{-1}t_{k}^{T}$$

where $P_{L_{k}}$ and $P_{I_{f\left(k\right)}}$are the associated covariance matrices, *s* and *t* are the stacked residuals vectors, the ${}_{W}^{I_{0}}\rho$ and ${}_{I}^{L}\rho$ are transform parameters can be computed as:

(33) $$\left\{ \begin{array}{l} s_{k}={}_{L_{k}}^{W}\rho -{}_{L_{k}}^{W}\hat{\rho }\\ t_{k}={}_{I_{f(k)}}^{I_{0}}\rho -{}_{I_{f(k)}}^{I_{0}}\hat{\rho } \end{array}\right.$$

We remark the ${}_{L_{i}}^{W}\rho$ and ${}_{I_{f\left(k\right)}}^{I_{0}}\rho$ are actually estimated quantities, but that we treat them as observations here. Considering the uncertainty in LiDAR and IMU measurements, we take that the residuals subjecting to *n* model constraints can be expressed as:

(34) $${}_{I_{f\left(k\right)}}^{I_{0}}\hat{\rho }={}_{I_{0}}^{W}\hat{\rho }\cdot {}_{L_{i}}^{W}\hat{\rho }\cdot {}_{I}^{L}\hat{\rho }$$

By using Lagrange multipliers and incorporating constraints, differentiating and rearranging, we subjected the Equation (33) to the *n* constraints and derived from Equation (34), the following equation can be obtained:

(35) $$\left[\begin{array}{l} \Delta {}_{W}^{I_{0}}\rho \\ \Delta {}_{I}^{L}\rho \end{array}\right]={\left[\sum_{k=1}^{n}J_{k}^{}M_{k}^{-1}J_{k}^{T}\right]}^{-1}\left[\sum_{k=1}^{n}\Delta y_{k}M_{k}^{-1}J_{k}^{T}\right]$$

where $\Delta {}_{W}^{I_{0}}\rho$ and $\Delta {}_{I}^{L}\rho$ are the incremental updates to ${}_{I_{0}}^{W}\hat{\rho }$ and ${}_{I}^{L}\hat{\rho }$, respectively, for the present iteration, and Δ*y* is a stacked vector, $\Delta y_{i}={}_{I_{f\left(k\right)}}^{I_{0}}\rho -\left({}_{W}^{I_{0}}\hat{\rho }\cdot {}_{L_{k}}^{W}\rho \cdot {}_{I}^{L}\hat{\rho }\right)$. The matrix *M_k_* is the combined observation covariance matrix, $M=P_{I_{f\left(k\right)}}+H_{k}P_{k}H_{k}^{T}$, where *H_k_* represents the Jacobian matrix with respect to observation ${}_{L_{k}}^{W}\rho$:

(36) $$H({}_{W}^{I_{0}}\rho ,{}_{L_{k}}^{W}\rho ,{}_{I}^{L}\rho )=\left[\frac{\partial ({}_{W}^{I_{0}}\rho \cdot {}_{L_{k}}^{W}\rho \cdot {}_{I}^{L}\rho )}{\partial ({}_{W}^{I_{0}}\rho )},\frac{\partial ({}_{W}^{I_{0}}\rho \cdot {}_{L_{k}}^{W}\rho \cdot {}_{I}^{L}\rho )}{\partial ({}_{I}^{L}\rho )}\right]$$

According to Equation (36), the ICP algorithm requires an initial estimate between the LiDAR and IMU data and we will use simple manual measurements to estimate the relative orientation between the LiDAR and IMU.

### 4.2. Iterated Sigma Point Kalman Filter (ISPKF) Algorithm for Compensation Calibration Parameters

After the relative orientation and optimal time delay estimation for the LiDAR-IMU, the ISPKF algorithm is used for compensation of the time delay calibration parameters between the LiDAR and IMU measurements.

The ISPKF algorithm uses the process noise component to implement the incremental state vector and the state covariance matrix, as expressed in:

(37) $${\hat{X}}_{a}(t_{k})=\left[\begin{array}{c} \hat{X}(t_{k})\\ n(t_{k}) \end{array}\right]$$

where *n*(*t_k_*) is the noise vector in measurement process, ${\hat{X}}_{a}\left(t_{k}\right)$ is the augmented state vector. At time *t_k_*_−1_, shortly after the LiDAR and IMU measurement update, the enhanced state covariance matrix $P_{a}^{+}\left(t_{k-1}\right)$ and the enhanced state mean ${\hat{X}}_{a}^{+}\left(t_{k-1}\right)$ can be formed as:

(38) $$P_{a}^{+}(t_{k-1})=\left[\begin{array}{cc} P^{+}(t_{k-1}) & 0_{m\times 12}\\ 0_{12\times m} & Q_{c}(t_{k-1}) \end{array}\right]$$

(39) $${\hat{X}}_{a}^{+}(t_{k-1})=\left[\begin{array}{c} {\hat{X}}^{+}(t_{k-1})\\ 0_{12\times 1} \end{array}\right]$$

where the state vector ${\hat{X}}_{a}^{+}\left(t_{k-1}\right)$ has size *m* = 26 + 3*n* and $Q_{c}\left(t_{k-1}\right)$ is the continuous-time covariance matrix for the noise vector *n*(*t_k_*_−1_), when *n* calibration points are included, for the Cartesian and inverse calibration points parameterization, respectively. The scaled form is employed for unscented transform, which requires a scaling term:

(40) $$\lambda =\alpha^{2}(N+\beta )-N$$

The *α* parameter is used to control the sigma points spread with respect to the mean of the state; we usually set a small positive value for parameter *α*. In the a posteriori state distribution Taylor series expansion, the *β* parameter is used to correct the higher order term. We set *β* = 2 and minimize the joint Gaussian distribution fourth-order error.

We use the augmented state vector ${\hat{X}}_{a}^{+}\left(t_{k-1}\right)$ to generate a set of sigma points according to the following equation:

(41) $$\left\{ \begin{array}{l} \chi_{a}^{\left(0\right)}(t_{k-1})={\hat{X}}_{a}^{+}(t_{k-1})\\ \chi_{a}^{\left(l\right)}(t_{k-1})={\hat{X}}_{a}^{+}(t_{k-1})+{\left(S(t_{k-1})\right)}_{j},j=l=1,\cdots ,N\\ \chi_{a}^{\left(l\right)}(t_{k-1})={\hat{X}}_{a}^{+}(t_{k-1})-{\left(S(t_{k-1})\right)}_{j},j=1,\cdots ,N,l=N+1,\cdots ,2N\\ S(t_{k-1})=\sqrt{\left(\lambda +N)P_{a}^{+}(t_{k-1}\right)} \end{array}\right.$$

where $P_{a}^{+}\left(t_{k-1}\right)$ is the augmented state covariance matrix, ${\left(S\left(\cdot \right)\right)}_{j}$ expresses the *j*-th column of the matrix *S*. The weight values of the associated sigma point can be described as:

(42) $$\left\{ \begin{array}{l} W_{m}^{\left(0\right)}=\lambda /(\lambda +N)\\ W_{c}^{\left(0\right)}=\lambda /(\lambda +N)+(1-\alpha^{2}+\beta )\\ W_{m}^{\left(j\right)}=W_{m}^{\left(j\right)}=\frac{1}{2(\lambda +N)},j=1,\cdots ,2N \end{array}\right.$$

A single σ-point can be propagated by the enhanced nonlinear process model function *f_a_*. Over the time interval $t\in \left[t_{k-1},t_{k}\right)$, the a priori state estimate and covariance *t_k_* can be computed as:

(43) $$\left\{ \begin{array}{l} \chi_{k}^{\left(p\right)}=f_{a}\left(\chi_{a}^{\left(p\right)}(t_{k-1})\right),p=0,\cdots ,2N\\ {\hat{X}}_{k}^{-}=\sum_{p=0}^{2N}W_{m}^{\left(p\right)}\chi_{k}^{\left(p\right)}\\ P_{k}^{-}=\sum_{p=0}^{2N}W_{c}^{\left(p\right)}\left(\chi_{k}^{\left(p\right)}-{\hat{X}}_{k}^{-}\right){\left(\chi_{k}^{\left(p\right)}-{\hat{X}}_{k}^{-}\right)}^{T} \end{array}\right.$$

Through propagating each σ point through the nonlinear measurement model function *h*, we can determine the predicted measurement vector as follows:

(44) $$\left\{ \begin{array}{l} {\mathbb{Z}}_{k}^{\left(p\right)}=h\left(\chi_{k}^{\left(p\right)}\right),p=0,\cdots ,2N\\ {\hat{Z}}_{k}=\sum_{p=0}^{2N}W_{m}^{\left(p\right)}{Ζ}_{k}^{\left(p\right)} \end{array}\right.$$

Through computing the a posteriori state vector, the state covariance matrix, and the Kalman gain matrix, we can perform the state update as follows:

(45) $$\left\{ \begin{array}{l} P_{{\hat{Z}}_{k}{\hat{Z}}_{k}}=\sum_{p=0}^{2N}W_{c}^{\left(p\right)}\left({\mathbb{Z}}_{k}^{\left(p\right)}-{\hat{Z}}_{k}\right){\left({\mathbb{Z}}_{k}^{\left(p\right)}-{\hat{Z}}_{k}\right)}^{T}\\ K_{k}=P_{{\hat{X}}_{k}{\hat{Z}}_{k}}{\left(P_{{\hat{Z}}_{k}{\hat{Z}}_{k}}+R_{k}\right)}^{-1}\\ {\hat{X}}_{k}^{+}={\hat{X}}_{k}^{-}+K_{k}(Z_{k}-{\hat{Z}}_{k})\\ P_{k}^{+}=P_{k}^{-}-K_{k}P_{{\hat{Z}}_{k}{\hat{Z}}_{k}}K_{k}^{T} \end{array}\right.$$

where *R_k_* is the measurement covariance matrix for *Z_k_*, while $P_{{\hat{Z}}_{k}{\hat{Z}}_{k}}$ and $P_{{\hat{X}}_{k}{\hat{Z}}_{k}}$ are the predicted measurement covariance matrix and the state-measurement cross-covariance matrix, respectively.

## 5. Experiments and Discussion

**Figure 5:**
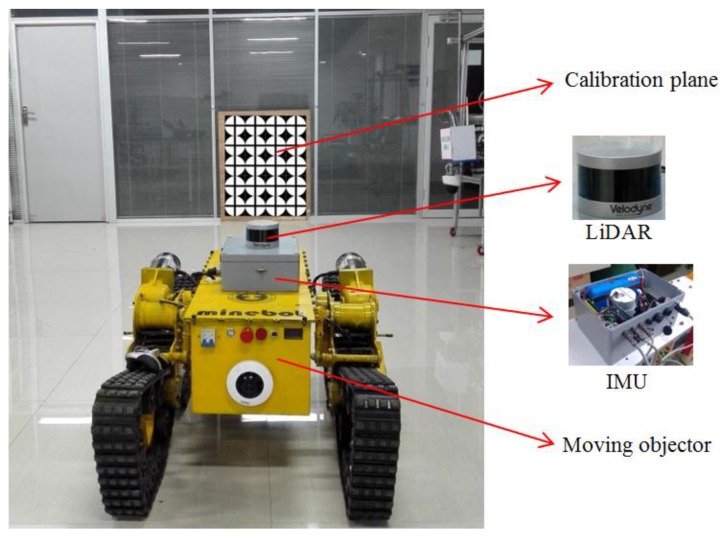
Experiment layouts.

To verify the effectiveness of the proposed algorithm, a field experiment was performed on the fourth floor of the School of Mechanical and Electrical Engineering building on the campus of China University of Mining and Technology. The experimental layout is shown in Figure 5. The experiments were conducted with a VLP-16 LiDAR (Velodyne, Morgan Hill, CA, USA) and Spatial FOG IMU (Advanced Navigation Company, Sydney, Australia). The specifications of the IMU and LiDAR are listed in Table 1.

The LiDAR consists of 16 laser scanners that collectively span a 27° of vertical field of view. Firstly, in each experiment, we initialize the IMU biases and keeping the IMU stationary for 3 s. We begin recording data from the LiDAR and the IMU, the moving objector carrying the LiDAR-IMU platform automatically moves following different configurations in front of the calibration plane. The IMU update data is recorded at 100 Hz, while the LiDAR scanning data rate is 100,000 points/s. We chose a set of three different trajectories for the LiDAR-IMU, and for each trajectory, we experimented with a different time shift and a different initial orientation estimate. In all trajectories, the initial true relative orientation of the IMU with respect to the LiDAR was the same. In the experiments, the moving objector ran three different trials, with the LiDAR-IMU platform translating and rotating in front of the calibration plane for approximately 5 s, and the LiDAR-IMU platform then ran in three different orientation and time delay configurations. For configuration 1, we set the IMU-camera relative orientation to a nominal value (a roll of 90°, pitch of 0°, and yaw of 90°), and fixed the estimated time delay value at zero. For configuration 2, we used the mean LiDAR-IMU relative orientation computed by averaging the roll, pitch, and yaw values, while again fixing the time delay at zero. Finally, for configuration 3, we used the averaged LiDAR-IMU relative orientation and the mean time delay value from configuration 2, and we determined the performance of each configuration by computing the RMS error. Once the LiDAR and IMU measurements for each configuration of the calibration plane were available, we used the method described in Section 4 to accurately estimate the LIDAR-IMU time delay calibration parameters and the LIDAR-IMU transformation parameters.

**Figure 6:**
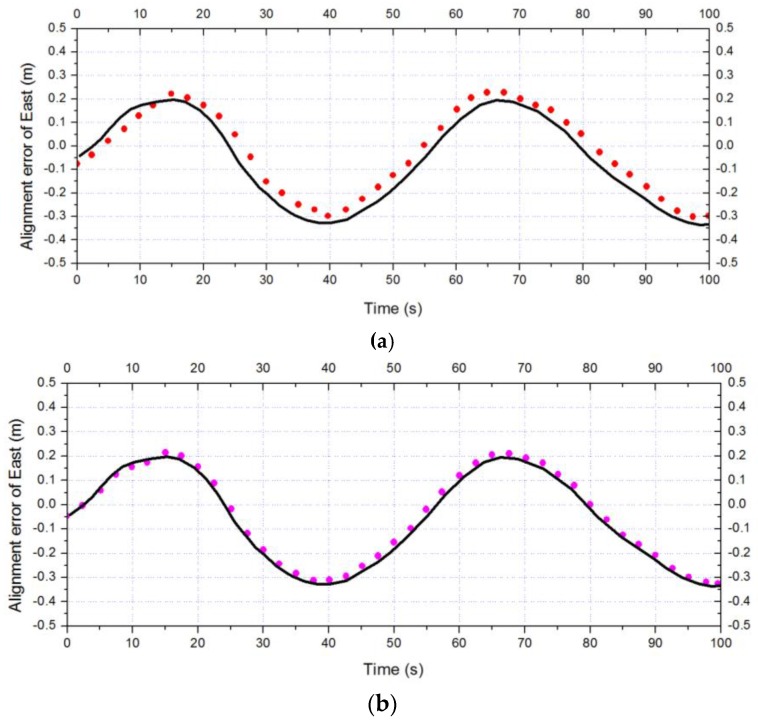

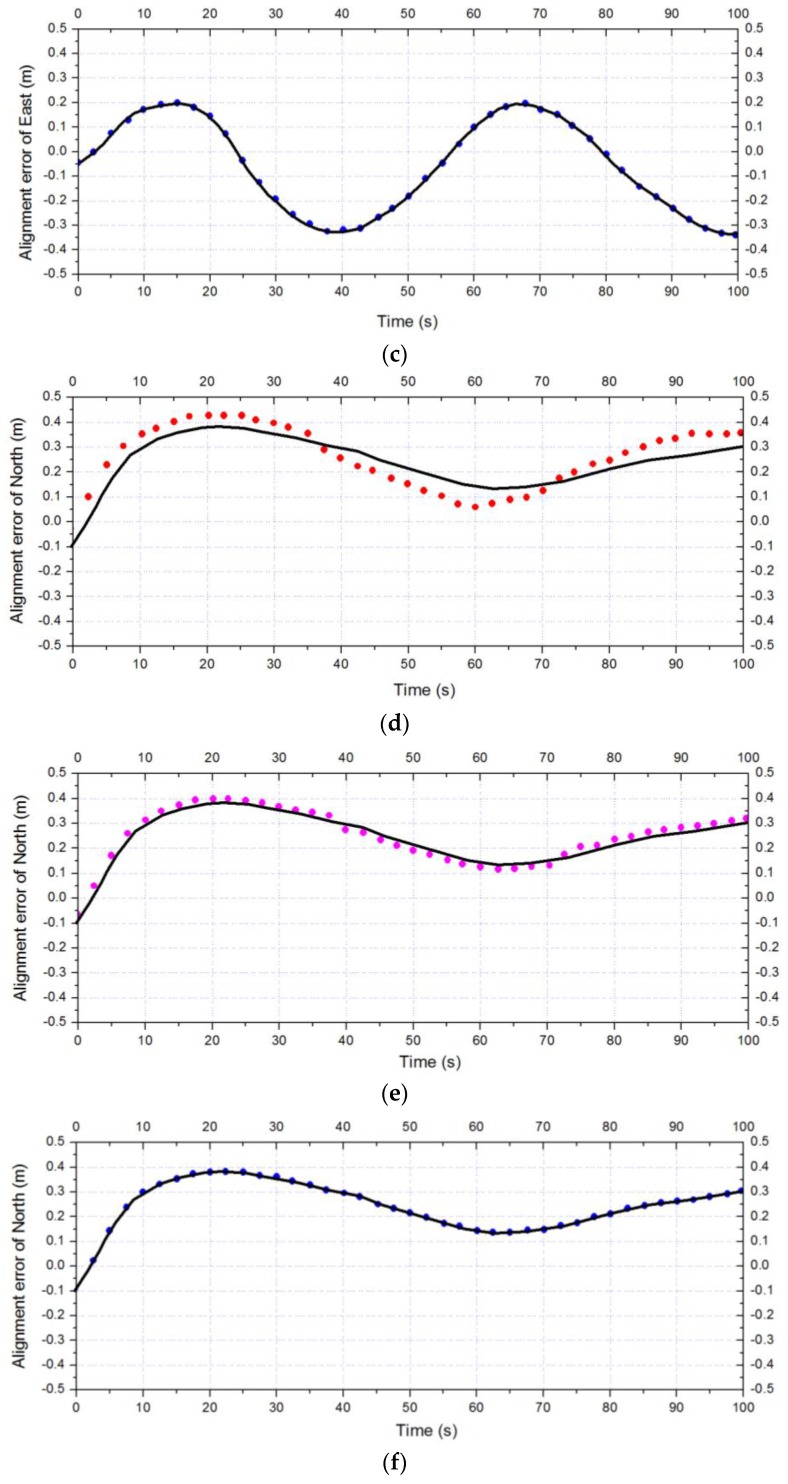

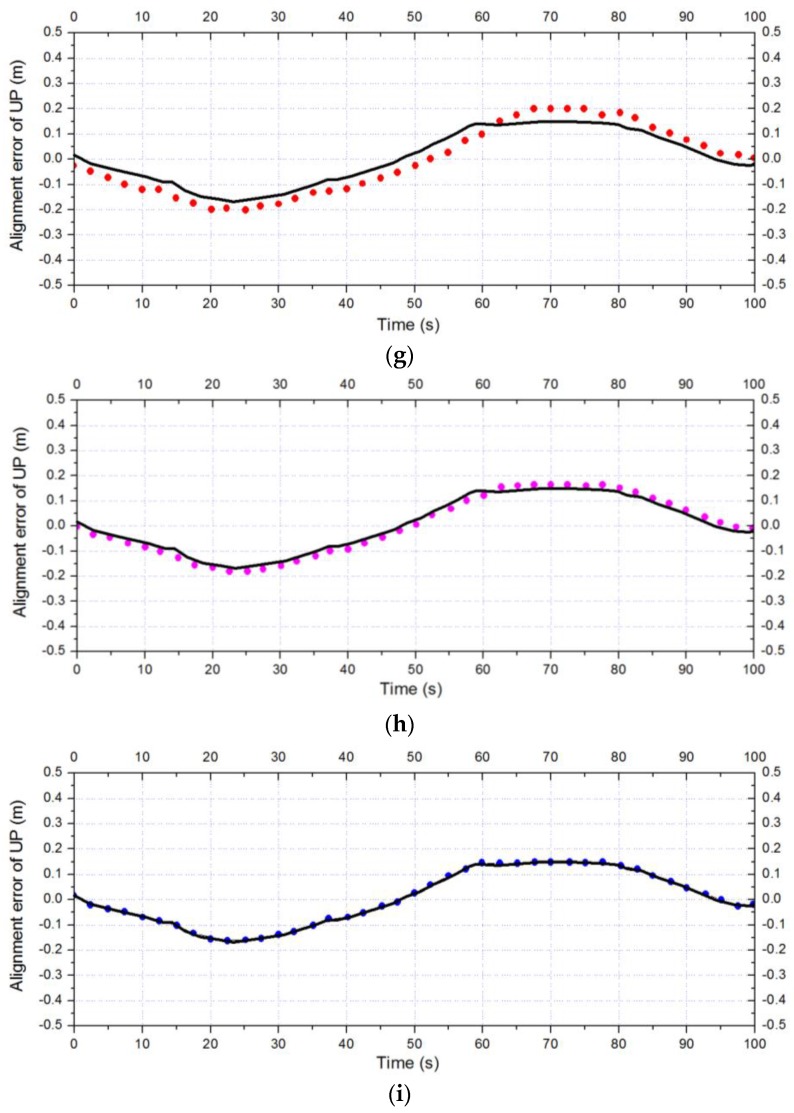
Time delay calibration using ICP-ISPKF for LiDAR-IMU. **(a**) Initial alignment between LiDAR (red dots) and IMU(black line) in East orientation; (**b**) time delay calibration one time using ICP-ISPKF between LiDAR (magenta dots) and IMU(black line) in East orientation; (**c**) time delay calibration ten times using ICP-ISPKF between LiDAR (blue dots) and IMU(black line) in East orientation; (**d**) initial alignment between LiDAR (red dots) and IMU(black line) in North orientation; (**e**) time delay calibration one time using ICP-ISPKF between LiDAR (magenta dots) and IMU(black line) in North orientation; (**f**) time delay calibration ten times using ICP-ISPKF between LiDAR (blue dots) and IMU(black line) in North orientation; (**g**) initial alignment between LiDAR (red dots) and IMU(black line) in Up orientation; (**h**) time delay calibration one time using ICP-ISPKF between LiDAR (magenta dots) and IMU(black line) in Up orientation; (**i**) time delay calibration ten times using ICP-ISPKF between LiDAR (blue dots) and IMU(black line) in Up orientation.

The experimental results for LiDAR-IMU time delay calibration as shown in Figure 6. Figure 6a,d,g, shows the initial alignment between IMU and LiDAR in East, North and Up orientation respectively. Figure 6b,e,h, shows one time alignment using ICP-ISPKF between IMU and LiDAR in East, North and Up orientation, respectively. Figure 6c,f,i, shows ten times alignment using ICP-ISPKF between IMU and LiDAR in East, North and Up orientation respectively. We examined the error in the LiDAR-IMU time delay calibration using ICP-ISPKF for ten times, as shown in Table 2.

According to the Table 2 and Figure 6, the initial time delay alignment error is 9.58 ms, the LiDAR-IMU alignment errors in the East, North and Up orientation are 0.093 m, 0.168 m, 0.089 m respectively. After one time calibration using ICP-ISPKF converged, the time delay alignment errors are reduced to 4.67 ms, and the alignment errors in the East, North and Up orientation are reduced to 0.067 m, 0.097 m, 0.063 m, respectively. For clarity, after ten times calibration using ICP-ISPKF converged, the final time delay alignment error was reduced to 0.50 ms, and the final alignment errors in the East, North and Up orientation were reduced to 0.018 m, 0.019 m, 0.017 m, respectively.

In order to prove the efficiency and accuracy of the ISPKF method, we used the ISPKF, KF and EKF methods to estimate the LIDAR-IMU time delay calibration errors. Since obtaining the initial truth time delay value is very difficult, we supposed the initial time delay alignment errors between LiDAR and IMU are zero, when the LiDAR and IMU measurements for each configuration of the calibration plane were available, the ICP-KF, ICP-EKF and ICP-ISPKF method are used to perform the time delay calibration for LiDAR-IMU.

**Figure 7:**
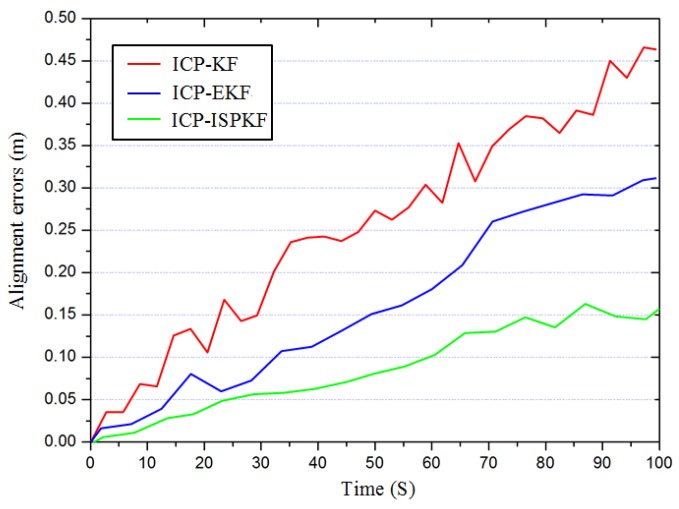
Time delay calibration using ICP-KF, ICP-EKF and ICP-ISPKF for LiDAR-IMU.

The experimental results are shown in Figure 7, where the red line is the time delay alignment error result using the ICP-KF method, the blue line is the time delay alignment error result using the ICP-EKF method, the green line is the time delay alignment error result using the ICP-ISPKF method, and the one time calibration mean alignment errors using the ICP-KF, ICP-EKF and ICP-ISPKF methods are 0.233 m, 0.151 m, 0.067 m, respectively.

## 6. Conclusions

In order to solve the problem of LiDAR-IMU time delay calibration, we have presented a fusion method based on an iterative closest point algorithm and an iterated sigma point Kalman filter, which combines the advantages of ICP and ISPKF. The ICP algorithm can precisely determine the unknown transformation between LiDAR-IMU; and the ISPKF algorithm can optimally estimate the time delay calibration parameters. We realized the coordinate transformation from the LiDAR frame to the IMU frame, and established the measurement model and time delay error model of LiDAR and IMU. We also presented the methodology of the ICP and ISPKF procedure for the LiDAR-IMU time delay calibration. Intensive experimental studies were conducted to check the validity of the theoretical results. The results show that after ten times calibration using ICP-ISPKF converged, the time delay alignment error was reduced from 9.58 ms to 0.50 ms, and the alignment errors in the East, North and Up orientation were reduced from 0.093 m, 0.168 m, 0.089 m to 0.018 m, 0.019 m, 0.017 m, respectively. In the future work, the ICP-ISPKF algorithm will be used to calibrate the time delay error in other multi-sensor systems. Within the batch execution framework, different methods will be examined for incorporating varying time delays. We are convince that our proposed methods will be useful for other types of time delay calibration for multiple sensors.

## Figures and Tables

**Table 1:** The specifications of the IMU and LiDAR.

IMU				LiDAR	
Navigation	Sensors Accelerometers Gyroscopes				
Horizontal Position Accuracy: 0.5 m	Range	10 g	490°/s	Channels	16
Vertical Position Accuracy: 0.8 m	Bias Instability	15 μg	0.05°/h	Range	100 m
Velocity Accuracy: 0.007 m/s	Initial Bias	<1 mg	<1°/h	Accuracy	±3 cm
Roll & Pitch Accuracy: 0.01°	Scaling Error	<0.03%	<0.01%	Vertical FOV	30°
Heading Accuracy: 0.05°	Scale Stability	<0.04%	<0.02%	Horizontal FOV	360°
Output Data Rate: Up to 1000 Hz	Non-linearity	<0.03%	<0.005%	Output Data Rate	300,000 pts/s

**Table 2:** Time delay calibration times using ICP-ISPKF for LiDAR-IMU.

Time Delay Calibration Times Using ICP-ISPKF	Time Delay Error (ms)	Alignment Error in East (m)	Alignment Error in North (m)	Alignment Error in Up (m)
0	9.58	0.093	0.168	0.089
1	4.67	0.067	0.097	0.063
2	2.45	0.043	0.068	0.041
3	1.66	0.037	0.047	0.035
4	1.17	0.031	0.039	0.029
5	0.87	0.026	0.032	0.025
6	0.63	0.023	0.027	0.021
7	0.57	0.020	0.024	0.019
8	0.53	0.019	0.021	0.018
9	0.51	0.018	0.020	0.018
10	0.50	0.018	0.019	0.017
