# LiDAR-IMU Time Delay Calibration Based on Iterative Closest Point and Iterated Sigma Point Kalman Filter. *Sensors* 2017, *17*, 539

**DOI:** 10.3390/s17122821

**Published:** 2017-12-05

**Authors:** Wanli Liu

**Affiliations:** School of Mechanical and Electrical Engineering, China University of Mining and Technology, Xuzhou 221116, China; lwl412101@cumt.edu.cn; Tel.: +86-516-8359-0706

The authors wish to make the following corrections to this paper [1]:

## 3.2. IMU Measurement Model

The IMU consists of three gyros and three accelerometers. The gyro provides change of Euler angles, while the accelerometers give the specific force. This model is based on the inertial measurement system error modeling method presented by Jonathan Kelly [5,27] and integrates the modified Rodrigues parameters kinematic equation. We obtained the IMU measuring equations as follows:

(18) $${\dot{P}}_{I}(t)=F(t)P_{I}(t)+P_{I}(t)F^{T}(t)+G(t)Q_{g}(t)G^{T}(t)$$

(19) $$F(t)=\frac{\partial \dot{\rho }(t)}{\partial v}=\frac{1}{2}\left(\left(\rho^{T}(t)\cdot \omega (t))I_{3}-{\left[\omega (t)\right]}_{\times }+\rho (t)\omega {\left(t\right)}^{T}-\omega (t)\cdot \rho^{T}(t\right)\right)$$

(20) $$G(t)=\frac{\partial \dot{\rho }(t)}{\partial n_{g}}=-\frac{1}{4}\left(\left(1-{\left\|\rho (t)\right\|}^{2})I_{3}+2{\left[\rho (t)\right]}_{\times }+2\rho (t)\rho^{T}(t\right)\right)$$

(21) $$\dot{\rho }(t)=\frac{1}{4}\left(\left(1-{\left\|\rho (t)\right\|}^{2})I_{3}+2{\left[\rho (t)\right]}_{\times }+2\rho (t\right)\right)\omega^{I}(t)$$

where $P_{I}(t)$ is the IMU orientation covariance at a time t, $Q_{g}(t)$ is an additive noise vector, ρ(t) is the modified Rodrigues parameters vector, F(t) and G(t) are the system matrix for ρ(t), ${\left[\rho (t)\right]}_{\times }$ is the skew-symmetric cross-product matrix for ρ(t), $I_{3}$ is the 3 × 3 identity matrix, $\omega^{I}(t)=\omega_{m}(t)-b_{g}-n_{g}(t)$ is the true angular velocity of IMU, and $b_{g}$ and $n_{g}(t)$ are the gyroscope bias vector and the noise vector, respectively. 

## 4.1. The ICP Algorithm for Estimation the Time Delay and Relative Orientation of LiDAR-IMU

The ICP algorithm is utilized to estimation the LiDAR-IMU time delay and relative orientation. At the beginning, the transforms between the LiDAR-IMU orientation curves are computed through iteratively selecting n correspondences point using the ICP algorithm. We employed the TD-ICP algorithm registration rules proposed by Jonathan Kelly [5,31] and by adjusting the search corresponding time scale and the orientation curves converge, this ICP algorithm can be described in two steps: 

Step I: Registration Rules.

The ICP algorithm operates by iteratively selecting the closest point between the IMU orientation curve and the LiDAR measurement point, and the concept of ICP proximity requires a suitable distance measurement. The minimum value of distance function can be computed as

(30) $$d_{ij}={(}_{L_{i}}^{W}\rho ,{}_{I_{j}}^{I_{0}}\rho )=4\arctan\sqrt{\sigma_{ij}^{T}\sigma_{ij}}$$

(31) $$\sigma_{ij}=-{(}_{W}^{I_{0}}\hat{\rho }\cdot_{L_{i}}^{W}\rho \cdot_{I}^{L}\hat{\rho })\cdot_{I_{j}}^{I_{0}}\rho$$

where $d_{ij}$ is the incremental rotation arc length taking from ${}_{L_{i}}^{W}\rho$ to ${}_{I_{j}}^{I_{0}}\rho$ in radians orientation, and $\sigma_{ij}$ is the spatial transform model on the unit sphere.

Step II: Nonlinear Iterative Registration Rules.

We can getncorrespondence relationship between IMU and LiDAR orientation measurement curves by selecting the closest IMU point for LiDAR point. The cost function used to align the IMU and LiDAR orientation curves can be calculated as

(32) U(ρWI0,ρIL)=∑k=1nskPLk−1skT+∑k=1ntkPIf(k)−1tkT

(33) sk=ρLkW−ρ^LkW

(34) tk=ρIf(k)I0−ρ^If(k)I0

where $P_{L_{k}}$ and $P_{I_{f(k)}}$ are the associated covariance matrices, s and t are the stacked residuals vectors, and the ρWI0 and ρIL are transform parameters.

By using Lagrange multipliers and incorporating the constraints ρ^If(k)I0=ρ^I0W⋅ρ^LkW⋅ρ^IL, differentiating and rearranging, Equation (32) can be minimized as

(35) U(ρWI0,ρIL)=[∑k=1nJk(PIf(k)+HkPLkHkT)−1JkT]−1[∑k=1n(ρ^If(k)I0−ρ^I0W⋅ρ^LkW⋅ρ^IL)Jk(PIf(k)+HkPLkHkT)−1JkT]

(36) H(ρWI0,ρLkW,ρIL)=[∂(ρWI0⋅ρLkW⋅ρIL)∂(ρWI0),∂(ρWI0⋅ρLkW⋅ρIL)∂(ρIL)]

where $H_{k}$ is the block-diagonal Jacobian matrix of the constraints with respect to ρLkW, and $J_{k}$ is the stacked Jacobian matrix of the constraints with respect to the transform parameters ρWI0 and ρIL.

The authors would like to apologize for any inconvenience caused to the readers by these changes.
